# Diagnostic and Interventional Role of Endoscopic Ultrasonography for the Management of Pancreatic Neuroendocrine Neoplasms

**DOI:** 10.3390/jcm10122638

**Published:** 2021-06-15

**Authors:** Giuseppinella Melita, Socrate Pallio, Andrea Tortora, Stefano Francesco Crinò, Antonio Macrì, Gianlorenzo Dionigi

**Affiliations:** 1 Department of Human Pathology of Adult and Pediatric Age, University of Messina, 98100 Messina, Italy; antonio.macri@unime.it (A.M.); gianlorenzo.dionigi@unime.it (G.D.); 2 Department of Internal Medicine and Medical Therapy, University of Messina, 98100 Messina, Italy; socrate.pallio@unime.it; 3 Digestive Endoscopy Unit, Azienda Ospedaliera Universitaria Policlinico, G. Martino, 98100 Messina, Italy; andreatortora11@gmail.com; 4 Gastroenterology and Digestive Endoscopy Unit, The Pancreas Institute, 37134 Verona, Italy; stefanocrino@hotmail.com

**Keywords:** pancreatic neuroendocrine neoplasms (PanNENs), endoscopic ultrasonography (EUS), endoscopic ultrasound-guided fine needle aspiration (EUS-FNA), endoscopic ultrasound-guided fiducial placement, endoscopic ultrasound guided tattooing, ethanol ablation, radiofrequency ablation (RFA)

## Abstract

Pancreatic neuroendocrine neoplasms (PanNENs) are relatively rare, but their incidence has increased significantly in the last decades. Precise diagnosis and prognostic stratification are crucial for proper patient management. Endoscopic ultrasound (EUS) is the modality of choice for diagnosis of solid pancreatic tumors, showing a higher tumor detection rate than other imaging modalities, especially for small size lesions. EUS also serves as a guide for preoperative sampling and other interventions. EUS-tissue acquisition is a safe and highly accurate technique for cyto/histological diagnosis of PanNENs with a well-demonstrated correlation between Ki-67 proliferation index values and tumor grading on EUS and surgical specimens according to the WHO 2017 classification. Furthermore, the possibility of a preoperative EUS-guided fine needle tattooing or fiducial markers placement may help the surgeon to locate small and deep tumors, thus avoiding formal pancreatic resections in favor of parenchymal-sparing surgery. Finally, locoregional ablative treatments using either ethanol injection or radiofrequency ablation have been proposed in recent studies with promising results in order to control symptoms or reduce tumor burden in selected patients unfit for surgery with functioning or non-functioning PanNENs. This article review highlights the current role of EUS in PanNENs management, focusing on the present and future applications of EUS-guided interventions.

## 1. Introduction

Pancreatic neuroendocrine neoplasms (PanNENs) are relatively rare (approximately 1 per 100.000 population annually) and account for only 1–2% of all pancreatic neoplasms [1,2]. The prognosis is far better than that of patients with adenocarcinoma because of their indolent and slow-growing tumor biology and their treatment strategy is consequently different from that of other pancreatic neoplasms [3]. They are classified as functional or non-functional depending on the presence or absence of a clinical hormonal hypersecretion syndrome [4]. Functional PanNENs (F-PanNENs) represent approximately 9% of all PanNENs and are classified according to the secreted hormone and subsequent functional syndrome: gastrinoma (4.2%), insulinoma (2.5%), glucagonoma (1.6%), and VIPomas (0.6%). Non-functional PanNENs (NF-PanNENs) (90.8%) are associated with no distinct clinical syndrome frequently secreting pancreatic polypeptide, chromogranin A, neuron-specific enolase, calcitonin, neurotensin, or other peptides [3,5,6,7,8]. Gastrinomas and insulinomas are the most common functioning PanNENs, with an estimated incidence of 1–3/million population/year [3,6,7,9,10,11]. Less than 10% are malignant. There is an age-specific incidence peak in the fifth decade of life and the incidence is slightly higher in women than in men. Approximately 10% are multiple, and 5% are associated with Multiple Endocrine Neoplasia 1 (MEN1) Syndrome [3,4,6,7,8,9,10,11,12,13]. Insulinomas located in the pancreas secret insulin, which causes symptoms secondary to hypoglycemic central nervous system effects (headache, confusion, visual disturbance, etc.) [4,6,7,10,11] or catecholamine excess (sweating, tremors, palpitation) [2,3,4,5,6,7]. The recent 2017 WHO classification (Table 1), based on the degree of differentiation and Ki-67 index, divides PanNENs into well-differentiated tumors (PanNETs), further divided into PanNETs-G1 (Ki67 index ≤ 3%), PanNETs-G2 (Ki67 index between 3% and 20%), and PanNETs-G3 (Ki67 index ≥ 20%), and carcinomas (PanNECs) which exhibit poorly differentiated morphology [14,15,16]. More than 90–95% PanNENs are benign at presentation and 90–100% can be surgically cured [3,4,6,10,12,15,16]. Tumor size >2 cm, Ki67 ≥3%, and various molecular features (chromosomal instability, chromosomal loss of 3p or 6q, chromosomal gain on 79, 12q or 14q) are all predictors of metastatic disease, which is associated with decreased survival [17]. Despite PanNENs still being considered rare [2,15,16], the widespread use of radiological imaging modalities and the advent of endoscopic ultrasound (EUS) have increased the incidental discovery of asymptomatic, small lesions exponentially over the last two decades [16,17].

The prognosis, therefore, depends on several factors such as the primary site, the histological classification as assessed by the specific WHO classification, mainly related to their proliferative activity, and the stage as evaluated by imaging and classified in a specific TNM system [12,13,14,15,16,17,18,19] (Table 1). The accurate localization of the primary tumor site as well as the correct staging of the discovered lesion together with a correct histological diagnosis have important prognostic implications and allow estimation of the progression and risk of recurrence after attempted curative resections [4,12,19,20,21,22,23]. Functional Pan-NENs localization is crucial before surgery but can be challenging due to their small diameter which is less than 10 mm in about 50% of the cases [24]. Many imaging modalities have been utilized, including trans-abdominal ultrasound (US), Computed Tomography (CT), and Magnetic Resonance Imaging (MRI). Indeed, data from 11 studies including 343 patients found an overall CT sensitivity of 73% (range: 39–94%) detection of PanNENs [25]. Similarly, a mean 73% (range: 50–94%) detection rate for PanNENs by using MRI was observed in 5 studies including 192 patients [25]. EUS provides high-resolution images of the pancreas and is regarded as one of the most accurate techniques for the diagnosis of pancreatic disease with a sensitivity ranging from 57% to 94% [12,26,27,28,29,30]. In the recently published European Neuroendocrine Tumor Society (ENETS) consensus guidelines, EUS has been considered the imaging study of choice to be performed after other non-invasive imaging studies are negative, allowing not only for the screening of the entire pancreas but also a detailed evaluation of the tumor [6,12,31,32,33]. A higher sensitivity was found by EUS compared with other cross-sectional imaging, with a mean detection rate of 90% (range 77–100%) in 10 studies comprising 261 patients [25]. Manta et al. [31] describe a large series of patients diagnosed with PanNENs in whom both EUS and CT were performed before pancreatic resection. CT showed an overall sensitivity of nearly 64% for detecting PanNENs, failing to detect lesions in more than 68% of those with a diameter of less than 10 mm and in a further 15% of patients with lesion diameter between 11 and 20 mm, while EUS was able to detect even very small tumors, including those lesions with a diameter ranging from 4 to 10 mm, which accounted for 47.5% in their series. The pathological characterization of the lesion, together with the accurate measurement of the diameter and location in the pancreas, confers to the pre-operative EUS procedure a very relevant role for PanNENs patient management. Therefore, EUS (detection and categorization) and CT (staging) should be considered complementary procedures to be performed in all patients with suspected PanNENs. It has been reported that when the results of CT are combined with EUS, a sensitivity of 100% can be achieved [31]. Moreover, in patients with F-PanNENs, important information that can be determined by EUS is the distance between the lesion and the main pancreatic duct, a factor that can determine the decision for the best surgical approach (i.e., enucleation versus resection) [6,12,34]. In addition, EUS-guided tissue acquisition (EUS-TA) is necessary to confirm the neuroendocrine nature of the pancreatic lesion and assess the grading of the neoplasia by determining the Ki-67 proliferation index, useful for the decision-making process and associated with 5-years survival of these patients [12,35,36,37,38]. Pre-operative knowledge of tumor grading may be crucial especially for small (≤2 cm) NF-PanNENs for which the choice between surgery and a clinical follow-up, besides patient preference, strongly depends on the tumor site and the value of Ki-67 proliferation index [39].

## 2. EUS Technique and B-Mode Evaluation

The intragastric and intraduodenal position of the EUS probe close to the pancreas allows the obtainment of high-resolution images and the visualization of local anatomic details. This peculiarity, together with the possibility of tissue acquisition has made EUS one of the most accurate tools for the evaluation of PanNENs [12,33,40,41,42].

**Figure 1:**
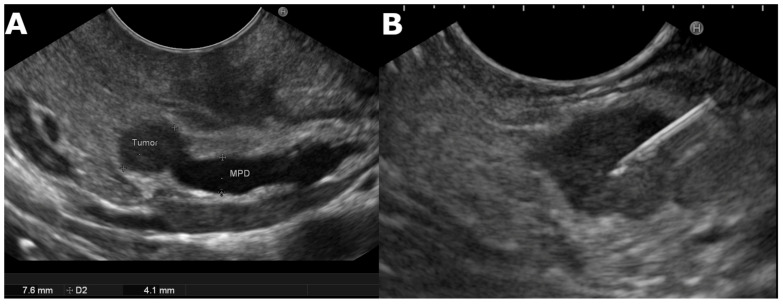
(**A**) Endoscopic ultrasound image of a small, well rounded, hypoechoic lesion with regular margin and dilated Wirsung in the head of the pancreas highly suggestive for neuroendocrine tumor. (**B**) The diagnosis of neuroendocrine tumor was confirmed by tissue specimen gathered using EUS-FNA. MPD, main pancreatic duct; D2, diameter.

Generally, a linear scanning echoendoscope is used, with the patient under deep sedation. The tip of the instrument is covered with a water-filled balloon allowing adequate transmission of ultrasound to improve image quality. A view of the whole pancreas is then taken according to the standard method of execution including a description of tumor location, size, margins (smooth or irregular), assessment of the echogenicity, distance from main pancreatic duct (MPD), evidence of upstream MPD dilatation, and vascular involvement [33,43]. PanNENs typically appear as hypoechoic, well-demarcated, round, hypervascular, homogeneous internal echo pattern lesions. [12,32,33,44,45] (Figure 1). The majority of PanNENs are solid lesions but less commonly may also appear as cystic [12,32,33,45]. However, lesions with diameter >2 cm, irregular margins, heterogeneous echotexture, and upstream dilation of the MPD are significantly associated with malignancy/aggressiveness completely indistinguishable from the most common adenocarcinoma [12,43,44]. In addition, because some patients may present multiple PanNENs, (MEN in 36% to 81%) [42] it is important to examine the entire pancreas to exclude synchronous lesions, and if present, awareness of suspicious features may help the endosonographer to choose the lesion to be punctured, [12,32,33,42]. The role of EUS stands out particularly in the evaluation of small neuroendocrine tumors (≤2 cm). Thanks to the improvement of resolution imaging diagnostic capabilities, pancreatic masses are often detected during routine cross-imaging studies. Several studies have shown that parenchymal-sparing surgery is effective and safe for patients with G1 and G2 PanNENs, and the key to the best therapeutic approach is an accurate pre-operative evaluation. EUS can provide detailed information about location, diagnosis, and grading, relationships with nearby structures, such as the main pancreatic duct and vessels, becoming an instrument for surgical enucleation [12,46,47,48]. This approach by ENET guidelines encourages parenchymal-sparing surgery, specifying how EUS is fundamental to therapeutic planning especially in asymptomatic and young patients [49,50]. Results from large studies including asymptomatic small pancreatic endocrine neoplasms will help to choose the best therapeutic strategy. EUS-TA with Ki-67 index evaluation could lead to personalized management of these patients in a multidisciplinary approach [45].

### 2.1. Endoscopic Ultrasound-Guided Tissue Acquisition

One of the most controversial issues in the diagnosis of PanNENs is the accurate prediction of their clinical behavior [51,52,53,54] and, according to the ENETS and WHO 2017 classifications, PanNENs grading should be expressed using the mitotic index and the Ki-67 proliferation index [17,55]. EUS-TA is nowadays considered the primary sampling technique for pancreatic tumors with a sensitivity ranging between 80% and 90%, a specificity close to 100%, and a sampling adequacy rate of 83–93% [31]. EUS-TA includes fine-needle aspiration (FNA) and fine-needle biopsy (FNB) (Figure 1). EUS-FNA is generally performed using standard 22-or 25-Gauge needles and provides cytological specimens. The choice of needle caliber depends on the diameter and site of the lesion, whether it is predominantly solid or cystic. In case of completely cystic lesions, to retrieve a piece of cystic wall, a microforceps able to pass through a 19 G needle has recently become available [56]. Since PanNENs are usually hypervascular, to reduce blood contamination of specimen, non-suction should be applied to the needle [57]. The samples are obtained as smears, right after the procedure [45]. The addition of rapid on-site evaluation (ROSE) can reduce the number of passes needed to obtain adequate material. In the absence of ROSE, ESGE guidelines suggest performing four FNA needle passes for the sampling of solid pancreatic lesions [58]. The overall complication rate of EUS-FNA (infections, bleeding, pancreatitis, duodenal perforation) is about 1–2% and is higher for cystic than solid lesions [33]. The overall adequacy of EUS-FNA for Ki-67 exceeded 84%, with a grading concordance between cytological and surgical specimens ranging between 58% and 78% [59]. A recent systematic review [60] showed a concordance rate of 83% for Ki-67 evaluation between EUS-FNA and surgical specimens.

Recently, the availability of needles specifically designed to obtain histological specimens is moving EUS-TA from EUS-FNA to EUS-FNB [61]. In particular, end-cutting needles represented a breakthrough in the field [61,62], and recent studies demonstrated EUS-FNB outperformed EUS-FNA for diagnosis of PanNETs, as for the preoperative evaluation of Ki-67 proliferative index [63,64,65]. In the study by Eusebi et al. [63], 91 patients with PanNENs sampled with EUS-FNA and/or EUS-FNB were included. The authors demonstrated a higher diagnostic sensitivity of EUS-FNB over EUS-FNA (94.3% vs. 88.4%) [63]. In another recent study, Leeds et al. compared outcomes of EUS-FNA and EUS-FNB in 57 patients with PanNENs [65]. A higher rate of adequate samples for the evaluation of the Ki-67 index was found in the EUS-FNB group (100% vs. 65.7%, *p* = 0.0006). Moreover, compared to surgical histology, Ki-67 on FNA correlated poorly (*r* = −0.08) whereas FNB correlated moderately (*r* = 0.65) [65]. Similarly, Crinò et al. [64] compared the yield of 51 EUS-FNA and 128 EUS-FNB samples from small (≤20 mm) NF-PanNETs. A significantly lower rate of Ki-67 feasibility was observed in the EUS-FNA group compared with the EUS-FNB group (88.2% vs. 96.1%, *p* = 0.04) [64]. However, valuable cytological specimens can be obtained also from specimens gathered with EUS-FNB using the touch imprint cytology [66,67]. Further studies are needed to investigate the potential complementary role of cytology and histology in the preoperative evaluation of PanNENs.

### 2.2. EUS Elastography

**Figure 2:**
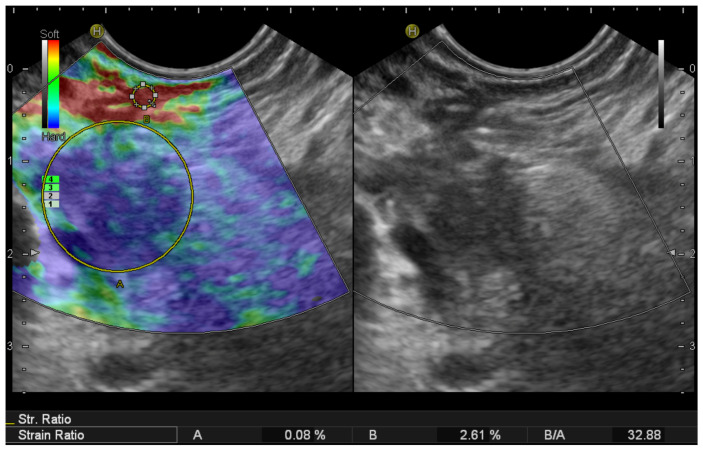
Endoscopic ultrasound elastography of a small pancreatic head neuroendocrine tumor. Two different areas (A, B) from the region of interest were selected for quantitative elastographic analysis. Area (A) is a lesion representative area including the biggest possible area of the tumor. Area (B) refers to soft (red) reference tissue outside the tumor. The quotient (B/A) (strain ratio) is considered as the measure of the elastographic evaluation.

EUS elastography (EUS-E) is a newer advancement in the field of diagnostic EUS for non-invasive characterization and differential diagnosis of solid pancreatic lesions [68]. Elastography evaluates tissue stiffness, which is different in normal pancreatic parenchyma, pancreatic cancer, and benign lesions [69]. Modern EUS image processors are equipped with software to assess the elastic properties of tissue which allow real-time elastography [70]. Malignant tissue tends to be harder than benign lesions or adjacent normal tissue [31,71]. EUS-E expresses the tissue stiffness in a qualitative form with different colors on the display, and a relative quantitative form that measures the tissue stiffness as strain ratio (SR) or strain histogram [69,70,71,72] (Figure 2). In patients with small solid pancreatic lesions, EUS-E can rule out malignancy with a high level of certainty if the lesion appears soft. However, a stiff lesion can be either benign or malignant. Because of the high negative predictive value (NPV) of EUS-E for the diagnosis of pancreatic ductal adenocarcinoma (PDAC) (98%), the finding of a soft pancreatic lesion <15 mm could potentially lead to deferral of surgical resection in a substantial subgroup of patients [71]. Iglesias-Garcia et al. [27] showed a sensitivity of 100% and specificity of 88% for quantitative elastography in differentiating PDAC from PanNENs when the cut-off value of strain ratio was 26.6. Carrara et al. [70] evaluated the role of quantitative EUS-E SR in the differentiation of small pancreatic lesions. Compared with benign lesions, malignant SPLs had a significantly higher SR (mean, 24.5; 95% CI, 19.8–29.2; *p* < 0.001). Pancreatic NENs had a significantly lower SR than malignant SPLs (7.1; 95% CI, 3.5–11.2; *p* < 0.001) but not significantly different from that of benign lesions (vs 5.4; 95% CI, 2.1–8.8; *p* = 0.441). Giovannini et al. [73] reported the sensibility and specificity of EUS-E in the diagnosis of malignant pancreatic lesions being 100% and 67%, respectively. Despite these encouraging results, the guidelines from the European Federation of Societies for Ultrasound in Medicine and Biology (EFSUMB) state that, to date, EUS-E cannot replace the cytopathologic diagnosis of pancreatic lesions, although it can guide further clinical decisions in case of inconclusive sampling [44]. Recently, shear-wave elastography (EUS-SWE) has been introduced as a real quantitative measurement examination on EUS. EUS-SWE is based on the properties of a shear wave and involves a Doppler-like ultrasound technique to monitor shear-wave propagation and to measure the velocity of the shear wave. Ohno et al. [73] evaluated the shear-wave velocity in 64 patients with solid pancreatic lesions including 43 PDAC, nine chronic pancreatitides, nine PanNENs, and three metastatic tumors. A significant difference in shear-wave velocity was not found based on the disease. However, in the same cohort, the authors observed that the mean strain elastography measured by strain histogram was significantly lower in patients with PDAC compared with those with chronic pancreatitis (45.4 vs. 74.5, *p* = 0.0007) [74]. Finally, EUS-E can be considered an additional diagnostic tool to select the much suspicious area of the pancreatic mass to be sampled [69,70,71,72,73,74,75,76].

### 2.3. Color Doppler EUS and Contrast-Harmonic EUS (CH-EUS)

**Figure 3:**
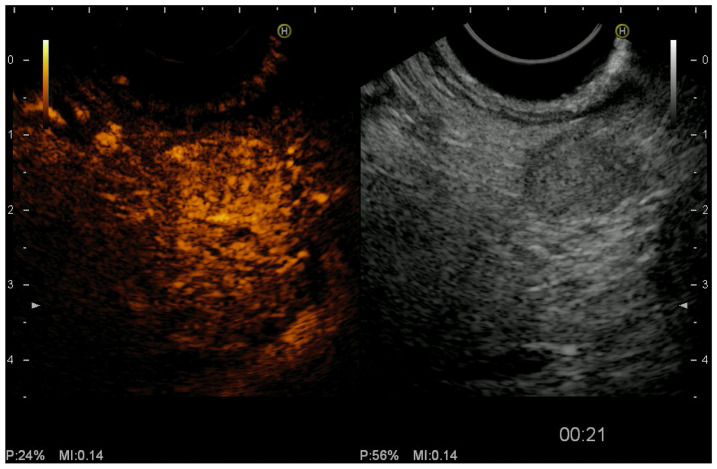
Endoscopic ultrasound (EUS) revealed a 10 mm hypoechoic lesion of the pancreas (**right panel**). Contrast-enhanced EUS showed typical homogeneous hyperenhancement of the lesion during the arterial phase in comparison to the surrounding pancreatic parenchyma (**left panel**) after intravenous injection of contrast agent (Sonovue™, Bracco Imaging, Milan, Italy). P, power; MI, mechanical index.

Color Doppler EUS evaluates the vascularity of pancreatic tumors [31]. Contrast-harmonic EUS (CH-EUS) is a technique that allows real-time visualization of microvasculature and pancreatic parenchyma perfusion, without Doppler-related artifacts, showing to be particularly useful in differentiating PDAC from other solid pancreatic lesions [31,76,77,78]. The development of microbubble-based contrast agents, together with advances in ultrasound technology, has led to improve imaging of fine vascular structures and visualization of microflow patterns within target lesions [76]. The most commonly used second-generation ultrasound contrast agents are composed of microbubbles of perfluoro gas entrapped into a lipid shell (e.g., Sulfurhexafluoride, Sonovue™, Bracco, Milan, Italy; Perfluorobutane; Sonazoid™, Daiichi-Sankyo, Tokyo, Japan; GE Healthcare, Milwaukee, WI, USA; not available in the USA and Europe). After having identified the target lesion, the second-generation contrast agent is injected as a bolus through an antecubital vein, using a 20-gauge catheter, followed by a 10/20 mL saline solution flush. The examination of the lesion lasts a minimum of 45 s after contrast agent injection [76,77]. The definition of ‘hypoenhancement’ is a pattern of enhancement in which the echo intensity of the lesion is lower than that of the surrounding pancreatic tissue on CH-EUS. It has been demonstrated that the hypovascular pattern predicts malignancy with a sensitivity of 92–96% and accuracy of 82–95% [79]. CH-EUS also provided greater diagnostic sensitivity (83.3%) for differentiating small PDAC (<2 cm) from other tumors when compared with power Doppler EUS (11%) and contrast-enhanced CT (50%) [80,81]. Kitano et al. [82] demonstrated the superiority of CH-EUS over other imaging modalities for diagnosing small PDAC (<2 cm), with sensitivity and specificity of 91.2% and 94.4% for CH-EUS and 70.6% and 91.9% for CT, respectively. On the other hand, hyper or isoenhancement is a strong negative predictor of PDAC. PanNENs are usually hyperenhancing (Figure 3). In the same study [82], the authors observed that hyperenhanced lesions were diagnosed as PanNETs with a sensitivity of 79% and specificity of 99%. PanNENs are a heterogeneous group of malignancies with different biological characteristics. Tumor size, grading, and the Ki67 proliferation index are known predictors of malignancy. Histologically, microvessel density is inversely correlated to tumor grading. The evaluation of microvascularization with CH-EUS has shown to have a high diagnostic value to predict malignancy with a sensitivity, specificity, and accuracy of 90.5%, 90%, and 90.2%, respectively [83]. The usefulness of CH-EUS in predicting malignancy of PanNENs has been evaluated in a recent retrospective study [84]. The authors included 92 patients with PanNENs and observed that heterogeneous enhancement on CH-EUS was able to predict malignancy with a sensitivity, specificity, PPV, and NPV of 93.3%, 93.5%, 87.5%, and 96.7%, respectively. A similar diagnostic yield was also shown in small (<2 cm) GI tumors. Palazzo et al. [77] demonstrated in a study how tumors with heterogeneous enhancement at an early arterial phase were likely to be aggressive. NPV value of CH-EUS for tumor aggressiveness was more than 95%. Diagnostic values were particularly high in the clinically relevant subgroup of G1/G2 non-functioning tumors without preoperative metastasis showing how CH-EUS should have a role in algorithm management in the choice between surgery versus the “wait and see” strategy and between oncology surgery versus ablation technique without lymph node excision. The differential diagnosis between benign and malignant lesions appears to be the main field of application of CH-EUS. CH-EUS may also predict PanNENs aggressiveness as tumors with heterogeneous enhancement have fewer vessels and more fibrosis which are the features associated with more aggressive tumors [84,85]. Concerning pancreatic solid neoplasms, the accuracy of CH-EUS seems comparable to that of EUS-TA. Moreover, the concomitant use of both CH-EUS and EUS-TA may prove to have additive value in increasing the overall accuracy by overcoming the false negative results of each individual technique. EUS-TA is not going to be replaced by CH-EUS; however, CH-EUS may contribute to the selective use of EUS-TA [80]. As a result of the reported advantages above, CH-EUS is the imaging modality of choice to discriminate PanNENs from PDAC at the first clinical assessment, particularly in the case of small tumors [76]. Further technical refinements and novel substances are likely to improve its diagnostic potential in the near future. Of particular interest, a quantitative assessment of CH-EUS has been proposed. Using dedicated software, temporal change of the echo enhancement intensity can be measured inside a region of interest (ROI) set with the maximum possible size in the center of the mass. As a result, a time-intensity curve can be achieved and different parameters evaluated (e.g., echo intensity change from baseline to peak, time for peak enhancement, the velocity of contrast imaging from baseline to peak, echo intensity reduction rate) [86]. In a study including 91 patients with different pancreatic diseases, the diagnostic accuracy based on the time-intensity curve was 88% and rose to 94.7% when considered in combination with EUS B-mode [86]. Moreover, Takada et al., in a study including 26 patients with PanNENs, demonstrated a diagnostic accuracy of time-intensity curve analysis close to 100% to differentiate G1/G2 PanNETs from G3 PanNECs [85].

## 3. Interventional Endoscopic Ultrasound for Pancreatic Neuroendocrine Tumors

### 3.1. EUS-Fine-Needle-Tattooing (EUS-FNT) and EUS-Guided Fiducial Implantation (EUS-FI)

Up to now, surgery, including both typical and atypical resections, has represented the mainstay treatment for PanNENs, with significant benefits in terms of survival [87], though curative pancreatic surgery is associated with significant short and long-term adverse events (AEs) [88]. A systematic review of the literature, including 62 studies, evaluating the most common postoperative complications in PanNENs, has reported that pancreatic fistula occurred in 45% of the cases after tumor enucleation, in 14% after both distal pancreatectomy and duodenopancreatectomy, and in 58% after central pancreatectomy. Delayed gastric emptying was observed in 5% of the patients after both enucleation and distal pancreatectomy, in 18% after duodenopancreatectomy, and in 15% after central pancreatectomy. Postoperative hemorrhage occurred in 6% of the cases. The overall pooled in-hospital mortality was 4% in distal pancreatectomy, 6% after duodenopancreatectomy, and 4% in central pancreatectomy [89]. Moreover, small lesions can be difficult to detect at the time of surgery [90]. In particular, up to 67% of pancreatic head insulinomas are non-palpable, and surgical resection in this area could become problematic, resulting in prolonged surgical time, increased risk of pancreatic duct injury, and consequently the choice of a duodenopancreatectomy [91,92].

Patients with small tumors are ideal candidates for pancreatic-sparing procedures such as enucleation or central pancreatectomy which allows for greater preservation of pancreatic tissue and function than more extensive procedures such as duodenopancreatectomy or distal pancreatectomy [90,91,92,93]. Therefore, the interest in interventions to facilitate identification of small PanNENs during surgical exploration is growing [94]. The ability of EUS to accurately visualize even very small PanNENs has inspired endosonographers to perform tattooing or fiducial markers implantation close to or inside lesions to facilitate and speed up their identification and resection in the process of surgery, especially during laparoscopy in which the tactile function of the surgeon is lost [94]. The first successful case of EUS-guided fine needle tattooing (EUS-FNT) was reported in 2002 by Gress et al. [95] who injected 4 mL of diluted India ink (Permark, Inc., Edison, NJ, USA) into a 19 × 5 mm ^2^ insulinoma of the body-tail junction using a 22-gauge FNA needle. The lesion was easily seen at laparotomy. The same group reported successful EUS-FNT of a non-palpable insulinoma in the pancreatic head, using 2 mL of sterile carbon-based ink (Spot^®^; GI supply, Camp Hill, PA, USA), which allowed very precise surgical dissection with preservation of the pancreatic duct [91]. In a case series of 13 patients including six PanNENs, EUS-FNT was performed by marking the pancreatic parenchyma using the above-mentioned sterile carbon-based ink to facilitate laparoscopic distal pancreatectomy. All tattooed tissues were clearly visible at surgery [96]. EUS-FNT represents a useful technique for the preoperative localization and surgical planning of PanNENs. Further studies are needed to confirm the efficacy and safety of EUS-FNT and its impact on the rate of duodenopancreatectomy in favor of parenchyma-sparing resections. Generally, a 22G standard needle should be used for performing EUS-FNT to allow easy injection of the tattooing solution but avoid its dispersion. The needle is inserted inside the target lesion where a small amount of the tattooing solution should be slowly injected. As an alternative, the injection can be performed immediately near the tumor borders into the normal parenchyma. Care must be taken not to over-inject the tattooing solution since the compound can migrate into the peritoneum or the retroperitoneal space and create major discoloration, which could impair the intraoperative lesion localization and consequently surgical resection [97].

**Figure 4:**
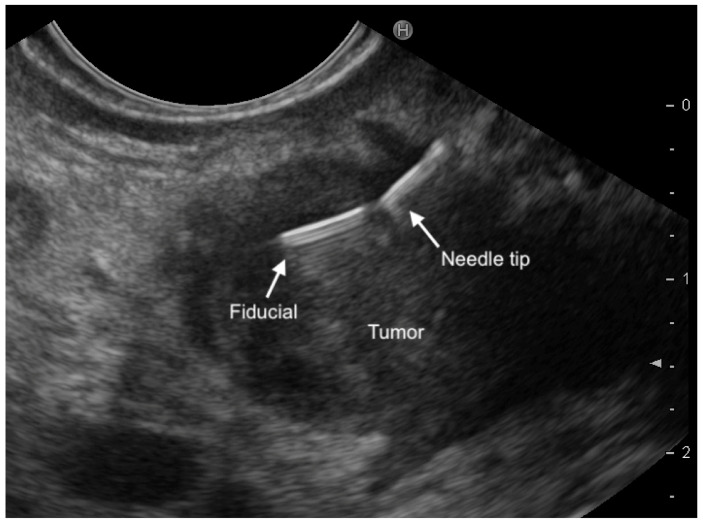
A magnified endoscopic ultrasound view demonstrating a fiducial just released inside a small pancreatic lesion.

Another EUS-guided technique to facilitate PanNENs localization during surgery is fiducial marker implantation (EUS-FI). Fiducials are implantable radiographic markers that have been used for identification of soft tissue in radiology. Traditionally, a gold fiducial (Visicoil; RadioMed Inc., Tyngsborough, MA, USA) with a length of 10 mm and two different diameters (0.75 or 0.35 mm) was used [98]. However, more recently, two specifically designed preloaded needles with a caliber of 22G have become available (EchoTip^®^ Ultra Fiducial Needle, Cook Medical, Limerick Ireland; Beacon™ FNF, Medtronic, Minneapolis, USA). The fiducial is easily visible within the tumor on EUS after deployment and during intraoperative ultrasound (Figure 4). In the last years, many studies have evaluated the safety and feasibility of EUS-FI in patients with pancreatic cancer. EUS-FI succeeded in two patients with small PanNENs [90]. The first was a 7 mm F-PanNET located in the uncinate process and the second a 9 mm NF-PanNET in the neck of the pancreas. Laparotomy was performed 14 days later. The lesion was easily identified using intraoperative ultrasound, and enucleation was performed. There were no procedure-related complications. In addition, there was no evidence of pancreatitis in the resected pancreas. Because the fiducial is placed either within or close to the lesion, EUS-FI offers very accurate tumor localization compared with EUS-FNT, which identifies the area but not the tumor. Both prospective and retrospective studies demonstrated the safety and technical feasibility of EUS-FI in solid pancreatic tumors. Further larger studies are required to determine whether the placement of fiducials not only aids identification but also decreases operative time [90]. Refinements in fiducial deployment are needed and the new multi-fiducial delivery system seems promising [98].

### 3.2. EUS-Guided Ethanol Ablation

Ethanol injection causes coagulation necrosis of tumor cells as a result of cellular dehydration, protein denaturation, and vascular occlusion. The technique has been widely used for image-guided percutaneous treatment of hepatocellular carcinoma [99,100]. Jurgessen and colleagues in 2006 first reported their successful experience of EUS-guided ethanol injection in a patient with insulinoma and symptomatic hypoglycemia, unwilling to undergo surgery. They used a total of 8 mL of 95% alcohol, injected into a 13 mm lesion. After treatment, although there were no further hypoglycemic episodes, the patient had mild acute pancreatitis which was resolved three days later. No tumor recurrence was noted after a 34-months follow-up [101].

Muscatiello et al. [102] reported a case of a patient with two pancreatic neuroendocrine tumors who refused surgical resection and underwent two sessions of EUS-guided ethanol ablation. Less than 2 mL of 40% ethanol was injected during each session. Although the first session was completed without complications, the second session was complicated by ethanol effusion with necrotizing pancreatitis requiring laparoscopic necrosectomy. At the two-months follow up, the somatostatin receptor scintigraphy and the biochemical exam for vasoactive intestinal peptide and chromogranin A were normal. Deprez et al. [103] reported a case of a 78-years-old female with insulinoma in the pancreatic head and symptomatic hypoglycemia treated with EUS-guided ethanol ablation after placement of a pancreatic and biliary stent. Complications included a mild and asymptomatic elevation of pancreatic enzymes for two days and the later occurrence of medically controlled hematoma and ulceration of the duodenal wall. Vleggaar et al. [104] reported a case of insulinoma of the pancreatic body with symptomatic hypoglycemia in an 82-year-old woman, a poor surgical candidate for heart failure, successfully treated with EUS-guided ethanol ablation. There were no symptoms of hypoglycemia up to six months after the ablation.

Levi and Topazian [105] reported a small series of 8 patients with symptomatic insulinoma managed by intralesional injection of 99% ethanol either under EUS (5) or intraoperative ultrasound guidance (8). There were no complications in the EUS-guided needle injection group. Park et al. reported successful EUS-guided ethanol injection in 11 patients with 14 PanNETs (10 non-functional and 4 insulinomas) who were poor surgical candidates [106]. Ten patients underwent clinical follow-up after treatment, and one patient was excluded because of loss to follow-up. A single session with an injection of 0.5 to 3.8 mL of ethanol resulted in complete response at the 3-month radiological imaging in seven out of 13 tumors (response rate, 53.8%). Multiple treatment sessions were carried out in three tumors with residual tissue. Mild pancreatitis occurred in three patients [106]. A single larger cohort study involved 39 pathologically confirmed small PanNETs (<2 cm) in high surgical risk patients. In this study, the authors used a solution of 99% ethanol and lipiodol, thus reducing the ethanol concentration, and observed a reduction of the AEs rate (3.2%). Complete ablation was achieved in 60% of cases. After a follow-up of 42 months (range 39–46), most G1 lesions showed a regression in size [107]. Technically, a EUS-FNA needle (22 or 25G) is advanced into the tumor and a very small volume of ethanol is injected (0.1 mL or less). The injections may be incrementally repeated at the same site until a hyper-echoic blush is visible expanding within the tumor. For larger lesions, multiple injections at different sites should be performed to cover the entire tumor and repeated sessions should be considered [108,109]. In terms of therapeutic outcomes, based on the literature review, this technique is feasible, relatively safe, and efficient. For small, functioning symptomatic G1 tumors, the aim of the ablation is symptom relief. The ethanol ablation treatment goal, for non-functioning tumors, is the complete ablation of the lesion confirmed by radiologic imaging [108,109,110]. Although published studies on EUS-guided ethanol ablation of PanNENs showed high success and low procedure-related complication rate, this technique should be reserved for patients who are poor surgical candidates or refuse curative resection [102,105,107,108,109,110]. Since previous studies have been comprised mostly of case reports, together with case series and pilot studies, multicenter controlled studies with long-term follow-up are needed to definitely prove the safety and efficacy of this procedure. Moreover, further refinements are necessary regarding optimal needle size, volume and optimal concentration of ethanol injection, the technique of EUS-guided ethanol ablation, and assessment of the treatment response to maximize efficiency and minimize AEs.

### 3.3. EUS-Guided Radiofrequency Ablation (EUS-RFA)

Radiofrequency ablation (RFA) is a locoregional treatment modality recently proposed for patients with both F-PanNENs and NF-PanNENs. It uses a high-frequency alternating current that generates thermal energy and induces coagulation necrosis in the tissue [109,111,112]. Overall, four RFA devices for EUS-guided applications are currently available. The first is the Habib EUS-guided RFA probe (EndoHPB, EMcision UK, London, UK; recently purchased by Boston Scientific). It is a 220 cm long, 1 Fr (0.33 mm), monopolar catheter, that can be inserted through a regular 22-gauge FNA needle and used in combination with commonly available radiofrequency generators. The other three are needle-electrodes, and the most commonly used in literature is the one from Taewoong Medical (EUSRA, Taewoong Medical Co., Ltd., Gimpo-si, Geyonggi-do, South Korea), an 18G or 19G needle with a 140 cm long electrode lacking insulation over the terminal tip (available from 5 to 30 mm in length), used in combination with a dedicated RF current generator (VIVA RF generator which allows control of power and impedance), and an inner cooling system that circulates chilled saline inside the needle to avoid tissue charring during large volume ablations [108,109,113].

The needle-electrode is inserted under EUS guidance into the target lesion by crossing the minimum of normal pancreatic parenchyma and avoiding major vessels, pancreatic or bile ducts. The echogenic needle tip is positioned at the far end inside the lesion. The energy delivery (burn) is then applied after confirming the location of the tip of the needle electrode within the margin of the lesion on EUS. On activation, echogenic bubbles gradually start appearing around the needle. Additional passes can be made to ablate different areas within the same lesion [112,113]. Depending on the RFA device characteristic and lesion size, more than one session may be required to achieve complete ablation. The extent of the ablated area varies, based on the amount of power used, procedure duration, and active electrode type and length [114,115]. After the procedure, CH-EUS can be performed to evaluate residual enhancing neoplastic tissue and to assess the need for further ablation. No guidelines on the timing to perform follow-up are available, but one month after seems to be a reasonable period [108]. Armellini et al. first reported successful EUS-RFA ablation of a non-functional 2 cm PanNET located in the pancreatic tail in an elderly patient who refused surgical resection. An 18G EUSRA needle-electrode was used, and a single session was performed [116]. EUS-RFA has been successfully employed as a minimally invasive treatment for both F- and NF-PanNENs in several case reports and a few case series [114,115,116,117,118,119,120,121,122,123,124]. A total of 30 F-PanNENs (exclusively insulinomas) in 25 patients were successfully treated without the occurrence of AEs [114,115,116,117,118,119,120,121,122,123]. Three case series have explored the utility of EUS-RFA in NF-PanNENs [122,123,124]. In the first published case series of seven patients, [122] 13 sessions of EUS-RFA were employed achieving a complete response in five patients. Two of the patients (28.6%) developed abdominal pain and mild pancreatitis, respectively. In a second case series, 14 lesions (median size 13.1 mm; range 10–20) in 12 patients with NF-PanNENs were treated with EUS-RFA [123]. Six- and 12-months following treatment, complete response was observed in nine and 12 lesions (64.2% and 85.7%), respectively. Two patients, however, developed major AEs. The first patient developed mild acute pancreatitis. The second patient with a 12 mm NF-PanNET located in the neck of the pancreas 1 mm far from the main pancreatic duct, developed ductal stenosis that required stent placement seven days after the procedure [123]. A third case series included 18 patients who were either unfit for surgery or preferred RFA over other treatment modalities. Among the 11 NF-PanNENs patients, eight showed a complete radiological response, one no response, while in the remaining two patients, the response was not assessed. Two cases of mild pancreatitis requiring brief hospitalization occurred after EUS-RFA (2- and 3-days post-RFA) [124].

Despite these promising results, the available evidence is too limited to recommend EUS-RFA treatment to the large majority of patients with PanNENs. Although feasibility for pancreatic tumors is acceptable, its translation into clinical use requires wider experience with long-term results. Moreover, patients’ selection must take into account comorbidity and risk of postoperative death, life expectancy, tumor location, risk of postoperative fistula and postoperative morbidity, and risk of long-term exocrine and/or endocrine insufficiency [108]. EUS-RFA may be added to reduce tumor burden often with palliative intent. With refinement in technique, there may be even a possibility of tumor downstaging. Future studies investigating the impact of EUS-RFA on quality of life and survival are needed to confirm its use in clinical practice. EUS-RFA is currently efficient for “functional PanNETs” providing early relief of symptoms. Its safety profile requires further confirmation in a large cohort of prospectively enrolled patients, especially for NF-PanNENs, where AEs have been observed probably due to the need for complete ablation as compared to F-PanNETs [108,109,110,111,112].

## 4. Conclusions

EUS has a central role in the setting of PanNENs. It allows detection of the tumor when other non-invasive procedures have failed and can provide additional information (e.g., distance from main pancreatic duct and Ki-67 proliferation index) for proper therapeutic management (surgery, conservative approach, type of anti-tumor therapy in case of unresectable disease). Furthermore, the possibility of EUS-FNT or EUS-FI of small “deep” lesions may help surgeons to find the neoplasms and avoid major surgery. Finally, EUS-guided therapies (e.g., alcohol injection or RFA ablation) are under investigation and, shortly, could represent an alternative to surgery in high-risk patients with both F- and NF-PanNENs.

## Figures and Tables

**Table 1:** Classification and grading criteria for pancreatic neuroendocrine neoplasms according to the WHO Classification of Tumors of Endocrine Organs, fourth edition (2017).

Terminology	Differentiation	Grade	Mitotic Rate	Ki-67 Index
NET G1	Well differentiated	Low	>2/10 HPF	<3%
NET G2	Well differentiated	Intermediate	2–20/10 HPF	3–20%
NET G3	Well differentiated	High	>20/10 HPF	>20%
NEC G3	Poorly differentiated	High	>20/10 HPF	>20%

NET, neuroendocrine tumor; NEC, neuroendocrine carcinoma, WHO, World Health Organization, HPF, high power field.

## Data Availability

Not applicable.
